# Fatal Rapidly Progressive Encephalopathy Mimicking Depression: A Diagnostic Dilemma of Suspected Seronegative Autoimmune Encephalitis

**DOI:** 10.7759/cureus.105251

**Published:** 2026-03-15

**Authors:** Yuuki Tanaka

**Affiliations:** 1 Department of Neuropsychiatry/Psychiatric Complication Center, NHO Kyushu Medical Center, Fukuoka, JPN; 2 Department of Community Mental Health &amp; Law, National Institute of Mental Health, National Center of Neurology and Psychiatry, Tokyo, JPN

**Keywords:** case report, cerebrospinal fluid oligoclonal bands, depression, general hospital psychiatry, rapidly progressive encephalopathy, seronegative autoimmune encephalitis

## Abstract

The diagnosis of "seronegative" autoimmune encephalitis (AE) is challenging, particularly when patients present with psychiatric symptoms but lack definitive neurological signs or specific autoantibodies. A fatal case of suspected seronegative AE in a male patient in his 50s with no documented history of psychiatric illness was reported. The patient initially presented with depressive mood and apathy, leading to a diagnosis of major depressive disorder. Despite antidepressant treatment, he rapidly exhibited confusion, agitation, and catatonic features. Conventional diagnostic procedures, including head magnetic resonance imaging (MRI) and cerebrospinal fluid (CSF) cell counts, yielded no remarkable findings. However, he exhibited paroxysmal autonomic instability (tachycardia and fever) and diffuse slowing on electroencephalography. CSF analysis revealed positive oligoclonal bands (OCBs), while neuronal surface antibody panels were negative. A clinical diagnosis of suspected seronegative AE was made based on the subacute progression and inflammatory markers, despite the absence of classic focal neurological signs or MRI abnormalities. Aggressive immunotherapy, encompassing high-dose corticosteroids, intravenous immunoglobulin, and plasma exchange, did not demonstrate any improvement. He succumbed to respiratory failure on Day 100. This case underscores the difficulty in distinguishing seronegative AE from other rapidly progressive encephalopathies or primary psychiatric disorders. While OCBs may indicate inflammation, relying solely on non-specific markers in the absence of stringent diagnostic criteria can result in diagnostic uncertainty. In treatment-refractory cases, clinicians must maintain a broad differential diagnosis, including neurodegenerative and neoplastic conditions.

## Introduction

The expanding spectrum of autoimmune encephalitis (AE) presents a formidable diagnostic challenge at the intersection of neurology and psychiatry. AE conditions can closely mimic primary psychiatric disorders, manifesting as acute psychosis, catatonia, mood dysregulation, or rapid-onset cognitive decline. This diagnostic overlap is particularly significant in anti-N-methyl-D-aspartate (NMDA) receptor encephalitis [1-3]. A recent nationwide survey in Japan revealed that at least 18.9% of patients with this condition are admitted to psychiatric units in general hospitals. This highlights the essential role of psychiatrists as frontline diagnosticians of neuro-immunological disease [4]. While NMDA receptor encephalitis is increasingly recognized, a subset of patients presents with clinical features of AE but lacks identifiable neuronal autoantibodies, termed "seronegative" AE.

Diagnosing seronegative AE typically relies on the exclusion of other causes and the fulfillment of clinical criteria, such as those proposed by Graus et al. [5], which require the subacute onset of deficits, focal CNS findings, seizures, or specific MRI/cerebrospinal fluid (CSF) abnormalities. However, diagnostic uncertainty arises when patients present with profound neuropsychiatric deterioration but lack "hard" objective findings like pleocytosis or MRI signal changes. In such "gray zone" cases, psychiatrists and neurologists face a critical dilemma: whether to pursue aggressive immunotherapy based on soft signs (e.g., oligoclonal bands) or to reconsider alternative fatal etiologies [6,7].

A case of a middle-aged man who initially presented with symptoms indistinguishable from severe depression, rapidly progressing to a fatal encephalopathy, is presented. This report illustrates the diagnostic complexities of suspected seronegative AE when standard diagnostic criteria are not fully met and discusses the pitfalls of relying on non-specific inflammatory markers in the absence of pathological confirmation. This case highlights the extreme difficulty of diagnosing seronegative AE when early clinical presentations mimic primary psychiatric disorders and specific biomarkers are absent.

## Case presentation

**Figure 1:**
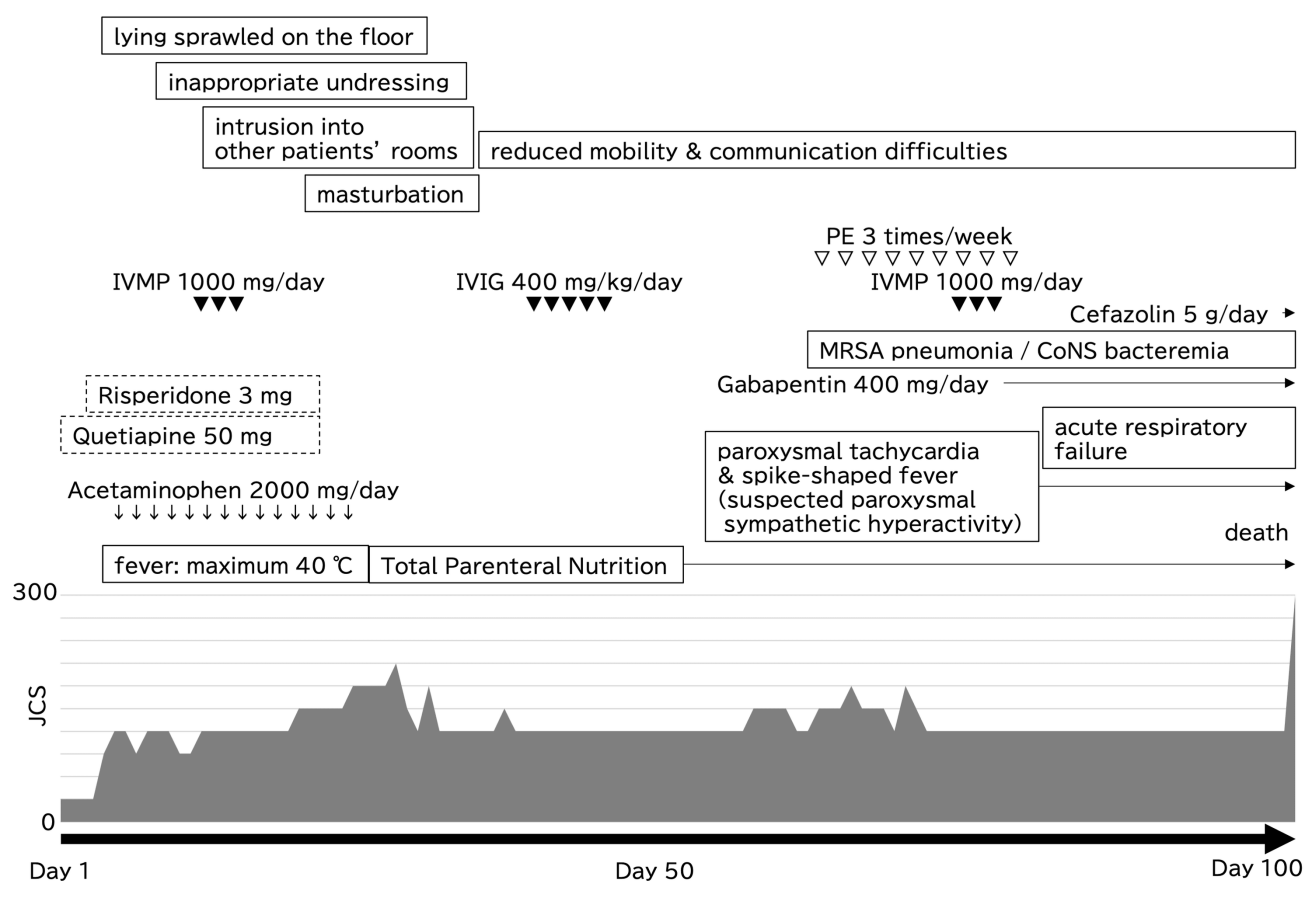
Timeline of the clinical course. The timeline summarizes the key events in the patient‘s clinical course, neuropsychiatric conditions, therapeutic interventions, fluctuation of consciousness (Japan Coma Scale: JCS [8,9]), and outcome. IVMP: intravenous pulse methylprednisolone, IVIG: intravenous immunoglobulin, PE: plasma exchange, MRSA: methicillin-resistant S*taphylococcus aureus*, CoNS: coagulase-negative S*taphylococci*.

A male in his 50s with no prior psychiatric history or family history of similar neurological or psychiatric illnesses presented to a psychiatric clinic with a depressive mood and loss of motivation, reportedly triggered by financial distress 180 days prior to admission. This identifiable psychological stressor strongly contributed to the initial misdiagnosis of a primary major depressive disorder, causing a diagnostic overshadowing effect. The overall clinical timeline is illustrated in Figure 1.

He was initially diagnosed with major depressive disorder and treated with pharmacotherapy (escitalopram 10 mg/day). Three months later (Day -90), he reported memory decline. A head magnetic resonance imaging (MRI) scan at a neurosurgery clinic revealed no abnormalities, and his symptoms were attributed to worsening depression. Subsequently, his speech and behavior became progressively bizarre, leading to his admission to our psychiatric ward on Day 1.

Upon admission, physical and neurological examinations revealed no gross focal deficits. Systematic neurological examination was largely unremarkable: cranial nerves (II-XII) were intact, and motor examination showed normal bulk, tone, and power (5/5) in all extremities. Sensory examination to pinprick, light touch, and vibration was intact. Cerebellar examination (finger-to-nose and heel-to-shin) showed no ataxia. Deep tendon reflexes were 2+ and symmetric globally, with absent pathologic reflexes (e.g., negative Babinski sign). There was no nuchal rigidity, and special maneuvers did not elicit startle myoclonus. However, cognitive assessment using the Japanese version of the Montreal Cognitive Assessment (MoCA-J) indicated significant impairment, with a total score of 15/30 [10]. Detailed subscores demonstrated broad cognitive decline, including visuospatial and executive functioning (2/5), naming (2/3), attention (3/6), language (2/3), abstraction (1/2), delayed recall (3/5), and orientation (2/6).

**Figure 2:**
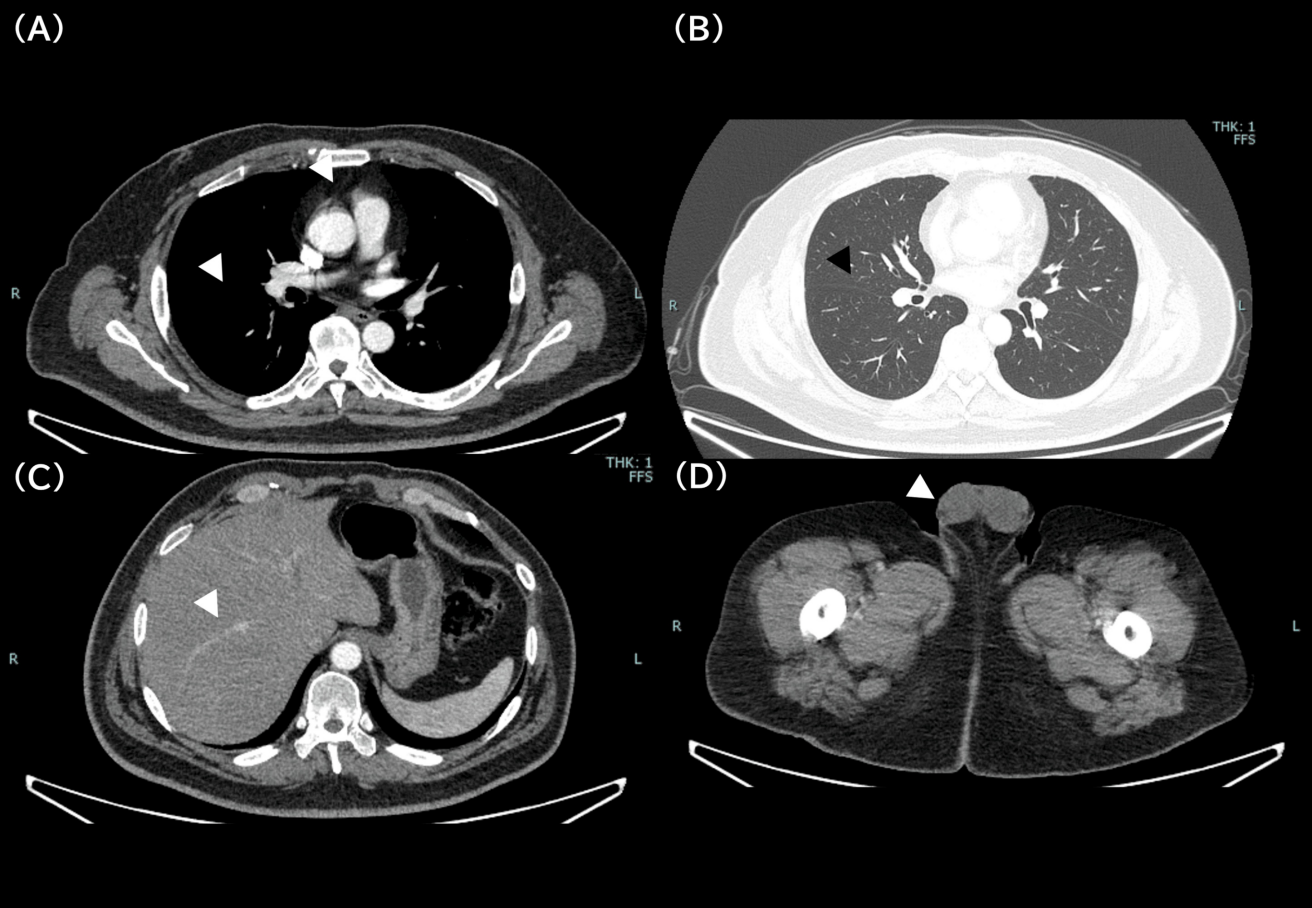
Whole-body computed tomography (CT) findings. Contrast-enhanced CT scans covering the chest (A, B), abdomen (C), and pelvis (D). The arrowheads indicate the normal-appearing lung fields, thymus, liver, and testes. These annotations are provided to explicitly demonstrate the absence of occult mass lesions in organs frequently associated with paraneoplastic neurological syndromes, such as thymoma or testicular germ cell tumors.

His mood was depressive with marked apathy. Routine blood tests, including thyroid function, adrenal function, syphilis, HIV, and inflammatory markers, were unremarkable. Whole-body contrast-enhanced computed tomography (CT) showed no malignancy (Figure 2).

**Figure 3:**
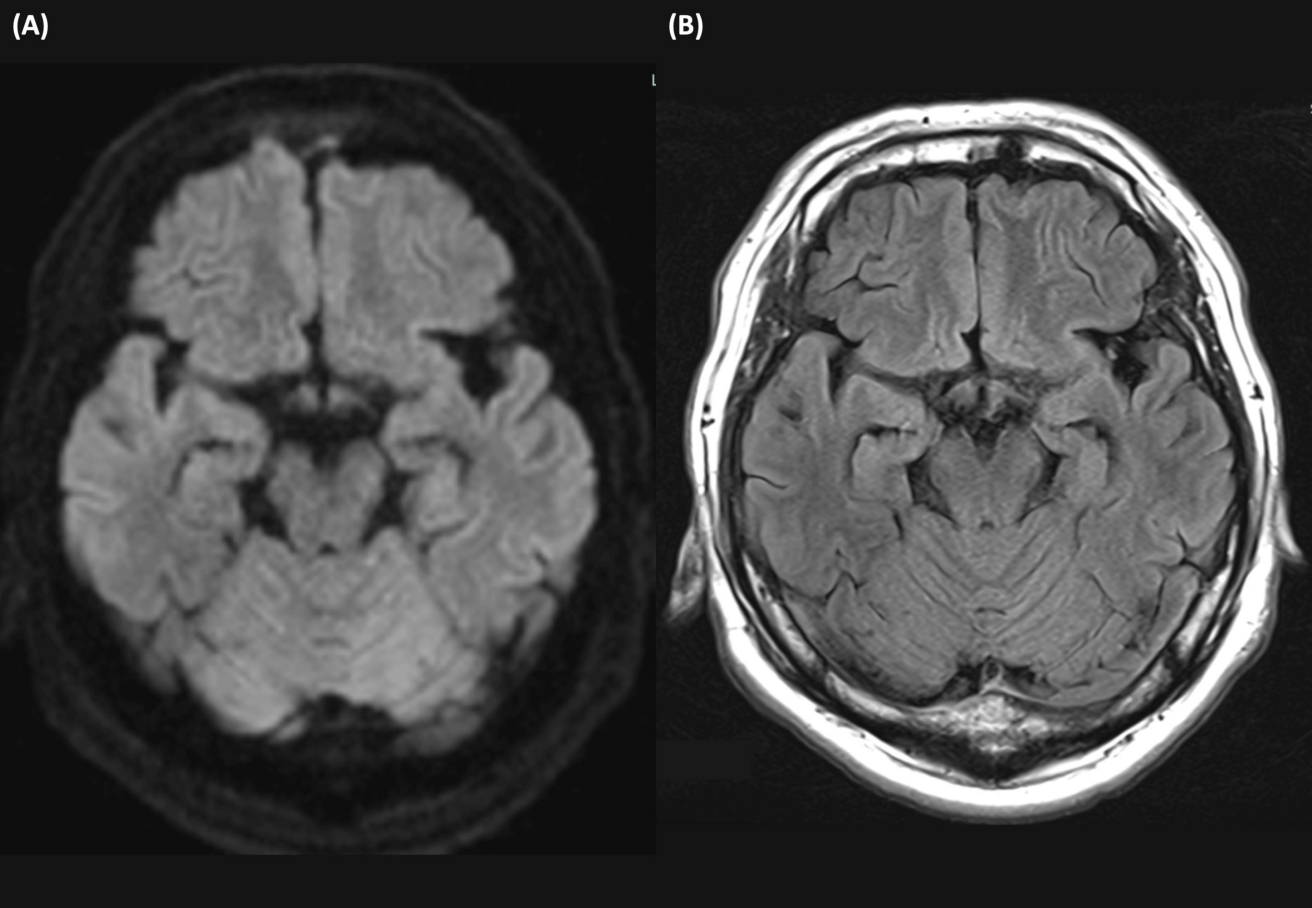
Brain magnetic resonance imaging (MRI) findings. Axial diffusion-weighted imaging (DWI) (A) and fluid-attenuated inversion recovery (FLAIR) imaging (B) obtained on admission. The scans demonstrate normal signal intensity and volume in the medial temporal lobes (hippocampi) and other regions.

**Figure 4:**
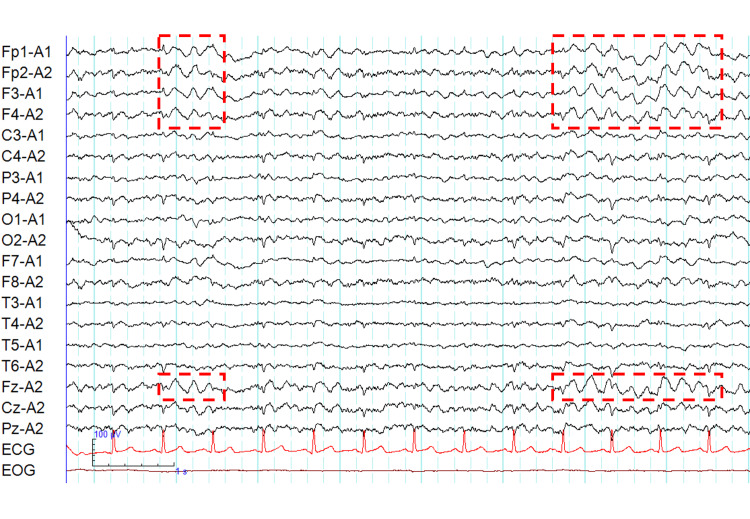
Electroencephalogram findings. The EEG (monopolar montage; sensitivity, 10 μV/mm; low-frequency filter, 0.53 Hz; high-frequency filter, 30 Hz; notch filter, 60 Hz) showed diffuse, slow-wave activity in the theta-delta range, replacing the normal background rhythm. The red dashed lines indicate areas of generalized slowing with a frontal predominance (Fp1, Fp2, F3, F4). No obvious epileptiform discharges, such as spikes or sharp waves, were observed. Crucially, there were no phase reversals, epileptiform discharges, or periodic synchronous wave complexes observed.

Despite optimizing psychotropic medications (the details of which are comprehensively provided in Figure 1), his condition deteriorated rapidly. He developed confusion, agitation, and reduced mobility. A repeat head MRI, including fluid-attenuated inversion recovery and diffusion-weighted imaging, showed no specific signal abnormalities, atrophy, or cortical ribboning (Figure 3). An electroencephalogram (EEG) on Day 6 (Figure 4) revealed diffuse delta and theta slow-wave activity without epileptiform discharges or periodic synchronous wave complexes.

CSF analysis on Day 7 showed normal cell count, protein, and glucose levels. Bacterial cultures and PCR for common viruses (HSV, VZV) were negative. A comprehensive panel for neuronal surface antibodies (including anti-NMDA receptor, anti-LGI1, and anti-CASPR2) was negative. CSF VDRL for syphilis was also negative. Assays for 14-3-3 protein and real-time quaking-induced conversion (RT-QuIC) were not requested; given the lack of a relevant family history and the perceived low probability of infection at the time of evaluation, prion disease was initially placed lower on the differential diagnosis. However, CSF-specific OCBs were positive.

During the clinical course, the patient exhibited paroxysmal tachycardia and spiking fevers (up to 40°C) without an infectious focus, along with generalized muscle weakness and mutism. Based on the subacute neuropsychiatric decline, diffuse encephalopathic EEG changes, autonomic instability suggestive of brainstem/hypothalamic involvement, and positive OCBs, a clinical diagnosis of suspected seronegative AE was made.

The patient was treated with three courses of high-dose intravenous methylprednisolone. Due to a lack of response and worsening consciousness (coma), intravenous immunoglobulin and five sessions of plasma exchange were administered. Third-line therapy with tocilizumab was considered due to the refractory nature of the disease; however, it was ultimately withheld owing to the concurrent severe infectious complications (MRSA pneumonia and CoNS bacteremia) and the patient's rapidly declining physiological reserve. Despite aggressive immunotherapy, the patient showed no clinical improvement. The course was complicated by aspiration pneumonia and sepsis, leading to death from respiratory failure on Day 100. An autopsy was not performed.

## Discussion

This case describes a fatal rapid-onset encephalopathy initially masquerading as major depression. The clinical trajectory highlights the formidable challenge of diagnosing "seronegative" AE, particularly when patients do not strictly fulfill established diagnostic criteria.

The diagnosis of seronegative AE typically relies on the criteria for "probable autoimmune encephalitis" proposed by Graus et al. [5]. According to these criteria, a diagnosis requires all three of the following conditions to be met: (1) subacute onset (rapid progression of less than three months) of working memory deficits, altered mental status, or psychiatric symptoms; (2) at least one of the following findings: new focal CNS findings, seizures not explained by a previously known seizure disorder, CSF pleocytosis, or MRI features suggestive of encephalitis; and (3) reasonable exclusion of alternative causes. In my case, the patient met the first criterion for subacute neuropsychiatric deficits. However, the fulfillment of the second criterion was ambiguous.

Standard MRI was normal, and CSF pleocytosis was absent. While the Graus criteria require "new focal CNS findings," I interpreted the patient’s paroxysmal sympathetic hyperactivity (tachycardia and high-grade fever) and generalized weakness as indicators of hypothalamic or brainstem involvement, supporting an organic etiology. Nevertheless, I acknowledge that these findings may not strictly satisfy the definition of focal neurological signs in the absence of classic deficits like hemiparesis or aphasia.

The diagnostic uncertainty in this case is a stark reminder of the challenges inherent in evaluating seronegative AE, particularly when early presentations mimic primary psychiatric disorders. When specific autoantibodies are absent and rigid diagnostic criteria (such as definitive MRI abnormalities or CSF pleocytosis) are not fully met, the diagnosis remains presumptive. In such instances, the differential diagnosis must be exhaustively broadened to include other rapidly progressive encephalopathies that can masquerade as treatment-refractory psychiatric conditions.

Foremost among these is Creutzfeldt-Jakob disease (CJD). CJD frequently presents with prodromal psychiatric symptoms, including profound depression and apathy, before progressing to rapid cognitive decline and myoclonus. While I deemed classic sporadic CJD less likely due to the absence of characteristic features in MRI and EEG, certain atypical subtypes (e.g., the VV1 or MM2-thalamic variants) can present with prolonged psychiatric features and initially unremarkable neuroimaging, making clinical differentiation from seronegative AE exceedingly difficult without advanced biomarkers like CSF RT-QuIC or tissue pathology [11, 12]. Furthermore, occult CNS malignancies, particularly primary CNS lymphoma variants such as lymphomatosis cerebri or intravascular large B-cell lymphoma (IVLBCL), must be strongly considered [13,14].

These conditions are notorious for causing rapidly progressive encephalopathy and behavioral changes without distinct contrast-enhancing mass lesions, sometimes showing only non-specific white matter changes or even normal initial scans. This diagnostic dilemma is further compounded by the empirical use of high-dose corticosteroids for suspected AE, which can transiently suppress lymphoma growth and alter radiological findings, thereby obscuring the true etiology. In the absence of a brain biopsy or autopsy, I cannot definitively exclude these fatal neurodegenerative or neoplastic mimics (i.e., atypical CJD or IVLBCL), highlighting the profound peril of relying solely on non-specific inflammatory markers like OCBs in the gray zone of neuro-immunology [15].

In the absence of specific biomarkers, the positive OCBs in the CSF served as the primary evidence for an inflammatory CNS process. While OCBs are non-specific and can be seen in various conditions including MS and chronic infections, in the clinical context of this patient, where infection and MS were excluded, they strongly supported an immune-mediated pathophysiology [7]. The initial presentation as major depression, with a normal neuroimaging result, likely contributed to a significant delay in considering an organic etiology, which may have adversely affected the patient's prognosis, as the efficacy of immunotherapy in AE is often time-dependent [16].

The limitations of this report include the lack of pathological confirmation via autopsy or brain biopsy. Without tissue diagnosis, atypical presentations of neurodegenerative disorders or occult malignancies cannot be definitively excluded.

## Conclusions

This case of fatal rapidly progressive encephalopathy, initially presenting as depression, serves as a critical reminder of the limitations of current diagnostic frameworks for seronegative AE. While the presence of CSF OCBs and autonomic instability prompted a diagnosis of suspected AE and subsequent immunotherapy, the absence of response and the fatal outcome suggest the need for caution.

Clinicians must maintain a high index of suspicion for organic etiologies in patients with atypical, treatment-refractory psychiatric symptoms. However, when specific autoantibodies are absent, and Graus criteria are not strictly fulfilled (e.g., normal MRI and CSF cell count), the diagnosis of seronegative AE should remain provisional. Crucially, this case underscores the absolute necessity of maintaining a broad differential diagnosis in patients presenting with rapidly progressive encephalopathy. A lower threshold for considering atypical prion diseases and occult CNS lymphomas is advocated and the urgent need for more specific biomarkers for seronegative autoimmune conditions is highlighted.
